# Dataset on the knowledge, attitude, and practices of biomedical waste management among Tehran hospital׳s healthcare personnel

**DOI:** 10.1016/j.dib.2018.08.002

**Published:** 2018-08-08

**Authors:** Mohammad Hadi Dehghani, Massuomeh Rahmatinia

**Affiliations:** a Department of Environmental Health Engineering, School of Public Health, Tehran University of Medical Sciences, Tehran, Iran; b Institute for Environmental research, Center for Solid Waste Research, Tehran University of Medical Sciences, Tehran, Iran; c Department of Environmental Health Engineering, School of Public Health, Iran University of Medical Sciences, Tehran, Iran

**Keywords:** Knowledge, Attitude, Health care waste, Hospital, biomedical waste

## Abstract

The data of this research was the investigation of knowledge, attitude and practices of biomedical waste management among healthcare staff and performed in some general hospitals in Tehran, Iran. In this descriptive data, 162 participants were chosen according to stratified sampling method and a self-made questionnaire was used for data collection. Also, Kruskal-wallis test, Mann -Whitney *U* tests and Spearman correlation coefficient were used to analyze the data in R software, version 3.4.4. The weighted mean of data showed that the knowledge level in staff is “Low” and their activity level is “Moderate”. Also, the data of the statistical analysis revealed that there is no significant difference between male and female health care personnel in knowledge, attitude and practices. However, the results of Kruskal-Wallis test showed that there was no significant difference between the views of hospital staff in occupational and educational groups about knowledge and attitude and their relationship with the history of passing the health course, while the difference in practices level was significant (*P* < 0.0001). Furthermore, the relation between attitude and practices level of participants with different work experience were not significant.

**Specifications Table**

**Table:** 

Subject area	Environmental Health Science
More specific subject area	Waste Management
Type of data	Table
How data was acquired	Data were collected by questionnaire
Data format	Raw, Analyzed
Experimental factors	The factors mentioned in the abstract were evaluated according to the completed questionnaires.
Experimental features	The researcher-made questionnaire, which contained data on Knowledge, Attitude, and Practices of Biomedical waste management among Healthcare Personnel were completed
Data source location	Tehran hospitals, Iran
Data accessibility	The data are available with this article

**Value of the data**

• The data showed a statistically significant positive relationship between Knowledge and years of service.
• The data is useful in showing that staff training is one of the fundamental ingredients in the field of proper management of biomedical waste.
• The data of the statistical analysis from this research can be useful as it indicates that it is necessary to hold some training course about biomedical waste management by relevant experts.

## 1 Data

Descriptive statistics related to the demographic information of the working personnel of case study hospitals were shown in Table 1. The data of Kruskal-Wallis test to compare the knowledge, attitude and practice of hospital staff regarding the management of hospital waste disposal in occupational groups was shown in Table 2, Table 3. Also, Table 4, Table 5 shows the data of the Mann-Whitney U test about the difference between the groups about the practices of hospital staff regarding the waste disposal management in occupational groups.Also, Table 6 shows the relationship between working personnel age, years of service and passing the health course with knowledge, attitude and practices. However, compare the range of scores for each field was shown in Table 7.

**Table 1:** Descriptive statistics related to the demographic information of the healthcare personnel.

**Variable name**	**Variable grouping**	**Knowledge level *N* (%)**	**Attitude rate *N* (%)**	**Behavior rate *N* (%)**	**Sum**	***X***^**2**^	**DF**	**p**
**Age(year)**	21–31	59(% 52.8)	59 ( % 52.7)	59(%52.7)	177(%52.4)	0.000	2	1
	31–41	32 (% 21.8)	32(%21.8)	32(%21.8)	92(%27.2)	0.08	2	0.96
	41–51	19 (% 16.7)	19(%16.7)	19(%16.7)	57(%16.9)	0.000	2	1
	51–54	4 ( % 3.5)	4(%3.5)	4(%3.5)	12(%3.5)	0.000	2	1
**Sex**	Female	117(%74.1)	117(%75)	116(%74.4)	350(%74.5)	0.006	2	0.99
	Male	41 (%25.9)	39(%25)	40(%25.6)	120(%25.5)	0.05	2	0.97
**Education level**	To diploma	36(%22.9)	34(%21.9)	35(%22.6)	105(%22.4)	0.06	2	0.97
	Associate Degree	17(%10.8)	17(%11)	17(%11)	51(%10.9)	0.000	2	1
	Bachelor	85(%54.1)	85(%54.8)	84(%54.2)	254(%54.3)	0.008	2	0.99
	Higher than bachelor	19(%12.1)	19(%12.3)	19(%12.3)	54(%12.2)	0.000	2	1
**Job**	Doctor	9(%5.8)	9(%5.8)	9(%5.8)	27(%5.8)	0.000	2	1
	Laboratory sciences	25(%16)	25(%16.2)	25(%16.2)	75(%16.1)	0.000	2	1
	Radiologist	19(%12.2)	19(%12.3)	18(%11.7)	56(%12.06)	0.04	2	0.98
	Paramedics and nurses	56(%35.9)	55(%35.7)	55(%35.7)	166(%35.8)	0.012	2	0.99
	services	29(%18.6)	28(%18.2)	29(%18.8)	86(%18.5)	0.023	2	0.98
	Technician	10(%6.4)	10(%6.5)	10(%6.5)	30(%6.45)	0.000	2	1
	others	8(%5.1)	8(%5.2)	8(%5.2)	24(%5.15)	0.000	2	1
**Years of service**	< 10	84(%59.6)	83(%59.7)	83(%59.7)	250(%59.65)	0.008	2	0.99
	10–20	43(%30.15)	42(30.2)	42(%30.2)	127(%30.3)	0.06	2	0.99
	20–30	14(%9.9)	14(%10.1)	14(%10.1)	42(%10.03)	0.000	2	1
**Passing health course**	Yes	71(%54.6)	70(%54.3)	70(%54.3)	211(%54.4)	0.009	2	0.99
	No	59(%45.4)	59(%45.7)	59(%45.7)	177(%45.6)	0.000	2	1

**Table 2:** Data of Kruskal-Wallis test about the knowledge, attitude and practices among healthcare personnel.

**Job Groups**		Doctor	Laboratory	Radiologist	Paramedics	Nurses	Health expert	Public Affairs	Services	Technician	Others	χ2	DF	Significant
**Variables**														
Knowledge	Number	9	25	19	6	50	4	3	26	10	8	17.957	9	0.036
	Average rating	10.94	8.84	9.58	4.83	62.38	9.38	102	88.42	93.8	84.13			
Attitude	Number	9	25	19	5	50	4	3	25	10	8	11.297	9	0.256
	Average rating	58.83	8.94	6.87	73	86.57	3.13	55.67	83.98	70.5	89.81			
Practices	Number	9	25	18	5	50	4	3	26	10	8	34.451	9	<0.0001
	Average rating	55	8.18	5.22	91.6	76.15	12.8	85.83	114.2	53.05	60.13

**Table 3:** Data of Kruskal-Wallis test about the knowledge, attitude and practice among educational groups regarding biomedical waste management.

**Variables**	**Study groups**	**Number**	**Average rating**	χ2	**DF**	**The significance level**

**Knowledge**	To diploma	36	81.38	3.787	3	0.290
	Associate Degree	17	76.15			
	Bachelor	85	74.7			
	Higher than bachelor	19	96.29			
**Attitude**	To diploma	34	73.21	3.867	3	0.176
	Associate	17	64.18			
	Bachelor	85	83.99			
	Higher than bachelor	19	72.13			
**Practices**	To diploma	35	100.06	11.743	3	0.008
	Associate	17	69			
	Bachelor	84	73.8			
	Higher than bachelor	19	64

**Table 4:** Data of the Mann-Whitney *U* test about the practices among healthcare personnel.

**Job Groups**	***Z***	**Significant Level**
Doctor with a health expert	-2.79	0.005
Doctor with services	-3.19	0.001
Laboratory sciences with services	-2.7	0.007
Laboratory sciences with Technician	-1.95	0.05
Radiology with Nurses	-2	0.04
Radiology with health expert	-2.72	0.003
Radiology with services	-3.91	< 0.0001
Radiology with health expert	-3.62	< 0.0001
Nurses with health expert	-2.41	0.016
Nurses with services	-3.62	< 0.0001
Health expert with Technician	-2.7	0.007
Health expert with others	-2.21	0.027
Services with Technician	-3.27	0.001
services with others	-2.59	0.011

**Table 5:** Data of the Mann-Whitney U for the difference between educational groups about the practices.

**Study groups**	***Z* Statistical**	**The significance level**
To diploma or Associate	-2.63	0.008
To diploma or Bachelor	-2.82	0.005
To diploma or Higher than bachelor	-2.76	0.006

**Table 6:** Spearman correlation coefficients between knowledge, attitude, practices, age, years of service and Passing the health course.

**Variables**		**Correlation rate**	**The significance level**
**Age**	Knowledge	0.156	0.097
	Attitude	0.108	0.256
	Practices	0.137	0.15
**Years of services**	Knowledge	0.199	0.018
	Attitude	0.087	0.307
	Practices	0.090	0.291
**Passing the health course**	Knowledge	0.21	0.89
	Attitude	0.434	0.28
	Practices	0.622	0.062

**Table 7:** Comparison the range of scores for each field.

**The range of scores for each field**	**Undesirable**		**Fairly Undesirable**		**Desirable**	
The scope of the study	Number	%	Number	%	Number	%
Knowledge rate	23	14.2	103	36.6	36	22.2
Attitude Status	3	1.9	1	0.6	156	96.3
Behavior Status	13	0.8	101	62.3	46	28.4

## 2 Experimental design, materials and methods

This survey-descriptive study was carried out in 5 university hospitals of Tehran to investigate knowledge, attitude and practices of healthcare staff on the appropriate handling and management of health care waste (HCW). 162 participants of personnel working in the wards of Tehran hospitals: doctors, nurses and service personnel participated in this study and the questionnaire was completed by them. The questionnaire included demographic questions: 10 questions about knowledge, 9 question about attitude and 11 question about practices [1], [2], [3], [4], [5], [6], [7], [8], [9], [10]. The validity and reliability of the questionnaire were tested by relevant experts in this issue and Cronbach׳s alpha equal to 0.78 was achieved. The knowledge questions were scored by order: 2 scores for “Yes”, 1 score for “No” and missing for “No idea” answer. The attitude and practices questions were scored by the Likert spectrum scaled from 1 to 5 score.
